# Perfumed Carbolic Acid

**Published:** 1881-02

**Authors:** 


### PERFUMED CARBOLIC ACID.

R. Acidi carbol. cryst., 1 part
Olei limonum, 3 parts
Alcoholis (36°), 100 parts. M.

This mixture, which appears to be quite stable and has only the odor of lemon, is what has been known as "Lebon's perfumed carbolic acid", the formula for which has long been a secret, but has now been made known in the *Moniteur Scientfique* of Paris. The antiseptic properties are in no way affected by the oil of lemon.

